# Vegetarian diets are associated with healthy mood states: a cross-sectional study in Seventh Day Adventist adults

**DOI:** 10.1186/1475-2891-9-26

**Published:** 2010-06-01

**Authors:** Bonnie L Beezhold, Carol S Johnston, Deanna R Daigle

**Affiliations:** 1 Department of Nutrition, Arizona State University, 6950 E. Williams Field Road, Mesa, Arizona, USA

## Abstract

**Background:**

The physical health status of vegetarians has been extensively reported, but there is limited research regarding the mental health status of vegetarians, particularly with regard to mood. Vegetarian diets exclude fish, the major dietary source of eicosapentaenoic acid (EPA) and docosahexaenoic acid (DHA), critical regulators of brain cell structure and function. Omnivorous diets low in EPA and DHA are linked to impaired mood states in observational and experimental studies.

**Methods:**

We examined associations between mood state and polyunsaturated fatty acid intake as a result of adherence to a vegetarian or omnivorous diet in a cross-sectional study of 138 healthy Seventh Day Adventist men and women residing in the Southwest. Participants completed a quantitative food frequency questionnaire, Depression Anxiety Stress Scale (DASS), and Profile of Mood States (POMS) questionnaires.

**Results:**

Vegetarians (VEG:n = 60) reported significantly less negative emotion than omnivores (OMN:n = 78) as measured by both mean total DASS and POMS scores (8.32 ± 0.88 vs 17.51 ± 1.88, *p *= .000 and 0.10 ± 1.99 vs 15.33 ± 3.10, *p *= .007, respectively). VEG reported significantly lower mean intakes of EPA (*p *< .001), DHA (*p *< .001), as well as the omega-6 fatty acid, arachidonic acid (AA; *p *< .001), and reported higher mean intakes of shorter-chain α-linolenic acid (*p *< .001) and linoleic acid (*p *< .001) than OMN. Mean total DASS and POMS scores were positively related to mean intakes of EPA (*p *< 0.05), DHA (*p *< 0.05), and AA (*p *< 0.05), and inversely related to intakes of ALA (*p *< 0.05), and LA (*p *< 0.05), indicating that participants with low intakes of EPA, DHA, and AA and high intakes of ALA and LA had better mood.

**Conclusions:**

The vegetarian diet profile does not appear to adversely affect mood despite low intake of long-chain omega-3 fatty acids.

## Background

Although adherence to vegetarian diets has been associated with physical health benefits, most notably a low risk of mortality from ischemic heart disease [1], vegetarian mental health is not well documented. Emerging evidence suggests that fish consumption has a protective effect on mental health due to the long-chain omega-3 fatty acid content [2]. Traditional vegetarian diets omit all flesh foods, and low intakes of the long-chain omega-3 fats, eicosapentaenoic acid [EPA] and docosahexaenoic acid [DHA], have been widely reported in vegetarians [3-6]. EPA and DHA favorably impact neural function by displacing the long-chain omega-6 fatty acids in brain cell membranes, particularly arachidonic acid [AA] [7]. AA is a key substrate for the synthesis of proinflammatory eicosanoids and downstream cytokines [8], which can adversely impact mental health via a cascade of neuroinflammation [9,10].

Vegetarians must rely on limited endogenous production of EPA and DHA from the short-chain omega-3 fatty acid, α-linolenic acid [ALA] in plant foods [11,12]. Yet, high intake of the plant-derived omega-6 fatty acid, linoleic acid [LA], characteristic of vegetarian diets [3-5], actually reduces omega-3 tissue incorporation [13-15]. A recent study reported the average omega-6 to omega-3 ratio in red blood cell phospholipids of vegans was 18.6 compared to 9.9 in omnivores [3], suggesting a higher overall inflammatory milieu for vegetarians. Research is needed regarding whether low omega-3 intake in vegetarians affects their mental health. The objective of this cross-sectional study was to compare the mood of vegetarians who never eat fish with the mood of their healthy omnivorous counterparts.

## Methods

Volunteers from Seventh Day Adventist (SDA) communities in the Phoenix, Arizona and Santa Barbara, California metropolitan areas in the United States were recruited for this study since they represent a particularly homogeneous group in terms of lifestyle characteristics [16]. Approximately one-third of Adventists eat no meat, fish, or poultry [17]. Individuals were excluded if pregnant or lactating, diagnosed with chronic disease affecting mental state, or regular users of medications or supplements known to influence mood. This trial was approved by the Institutional Review Board at Arizona State University. Sixty-four vegetarians and seventy-nine non-vegetarians read a letter of consent and completed an anonymous survey which took approximately 30 minutes. Completion of the survey indicated consent. The survey included three parts: a general health history questionnaire, a food frequency questionnaire (FFQ), and two psychometric tests, the Depression Anxiety Stress Scale (DASS) and the Profile of Mood States (POMS).

The healthy history questionnaire captured demographic information including a categorical question about education level completed, supplement and medication intake, and a validated total weekly leisure activity screening tool [18], since it is well established that physical activity level can modulate mood [19]. Participants were asked to report their weekly frequency of strenuous, moderate, and light intensity activities which were multiplied by nine, five, and three METs, respectively, and total weekly leisure activity was calculated by summing the products of the separate components. Multiple level education data was collapsed into two categories (college and no college) for analysis.

The FFQ was a 152-item quantitative questionnaire which was previously validated for use in estimating the intake of n-3 fatty acids in cardiac patients [20]. We modified the FFQ slightly with the addition of other foods commonly consumed by vegetarians, and in addition to estimating n-3 fatty acid intake, we estimated the intakes of other major fatty acids using the USDA's Food and Nutrient Database for Dietary Studies (United States Department of Agriculture, 2008). Participants indicated their portion sizes and intake frequency for each food item. The estimated intake of each type of fatty acid was calculated by multiplying intakes by the selected serving size: small (0.5), medium or standard USDA serving size (1.0), and large (1.5), and by the frequency of consumption selected: once a month, less than once a week, 1-2 times a week, 3-4 times a week, 5-6 times a week, daily, and more than once a day, and entered into the spread sheet as 1, 3, 6, 14, 22, 30, or 60, respectively. Estimated intake of each fatty acid type was calculated using Microsoft Excel 2007 software (Microsoft Corp, Seattle, WA).

The DASS was designed to measure three related but distinct negative affective states in nonclinical populations: depression (D) which assesses dysphoria, hopelessness, devaluation of life, self-deprecation, lack of interest, anhedonia, and inertia; anxiety (A) which assesses autonomic arousal, skeletal musculature effects, situational anxiety, and subjective experience of anxious affect; and stress (S) which assesses difficulty relaxing, nervous arousal, and being easily agitated, irritable, overreactive, and impatient [21]. This scale has been validated for use in nonclinical populations and in research settings [22,23]. The 42-item questionnaire contains 14 complete sentence items for each of the three areas, and subjects are asked to use 4-pt severity/frequency scales to rate the extent to which they have experienced each state over the past week, with higher scores indicating a greater degree of mood disruption. The DASS takes approximately 10 minutes to complete. The total score is determined by summing the three subscale scores (reported normative score for nonclinical population: 18.38) [24]. Reliability of the three scales is considered excellent, with Cronbach's alpha at .95 for D, .90 for A, .93 for S, and .97 for total score [23]. Test-retest reliability is also excellent with .72 for D, .79 for A, and .81 for S [22]. The DASS-D correlates with the Beck Depression Inventory (BDI-II; r = .74), the standard clinical measure of depression [21]. The DASS has adequate convergent and discriminant validity (CFI = .93) [23].

The POMS (Educational and Industrial Testing Service, San Diego, CA) estimates the intensity of mood disturbance, is easy to administer, and is one of the most widely used and accepted mood scales in healthy populations [25,26]. It consists of 65 adjectives rated on a 5-pt Likert-type scale ranging from 'not at all' to 'extremely', and covers six mood domains: tension-anxiety (POMS-T); depression-dejection (POMS-D); anger-hostility (POMS-A); vigor-activity (POMS-V); fatigue-inertia (POMS-F); confusion-bewilderment (POMS-C); the total POMS score was computed by summing the five negative domain scores (T, D, A, F, and C) and subtracting the vigor (V) score. Higher scores indicate a greater degree of mood disturbance (normative mean scores: males 14.8, females 20.3) [26]. These data are based on 'during the past week, including today' time frame. The POMS has a high degree of reliability, with reported Cronbach's alpha ranging from .84 to .95 [25]. Total POMS is moderately-to-highly correlated with standard scales (the Visual Analog Mood Scales, the State-Trait Anxiety Inventory, and the Beck Depression Inventory) with coefficients ranging from .72 to .79 [26].

Since vegetarians have a relatively higher prevalence of anemia due to lower intake of iron and vitamin B_12 _[27] and because this condition can adversely affect mood [28], the first half of our participants were tested for anemia (n = 63). A finger stick was administered by a trained phlebotomist, and hematocrit was determined from a drop of capillary blood.

Descriptive statistics were reported for all outcome measures and data are reported as mean ± SE. Independent sample t-tests and Chi-square tests were utilized to examine the impact of differences between group characteristics. Mood scores were normalized by square root transformation prior to analyses, and differences between groups were examined using independent sample t-tests and univariate ANCOVA when adjusting for confounding variables. Dietary data could not be normalized; the Mann-Whitney U test was utilized to examine differences between groups. Spearman's correlation was used to assess relationships between variables. Data were analyzed using The Statistical Package for the Social Sciences (SPSS, versions 15 and 16 for Windows, 2006, Chicago IL) and p values < 0.05 were considered significant.

## Results

### Participant characteristics

Data are reported for 138 participants, 107 participants were from the Phoenix SDA community and 31 were from the Santa Barbara SDA community. Mean population characteristics including mood scores did not differ by location. Five participants (one non-vegetarian and four vegetarians) of our initial survey population (n = 143) were removed from the data set prior to analyses due to either reported anti-depressant use (an exclusion criteria) or extreme reported total POMS score (> 3SD from the mean) leaving a total of 138 participants; and for the diet analysis only, three participants (all VEG) were excluded due to extreme dietary polyunsaturated fatty acid intakes. Mean age, BMI, education, and PA values for these eight excluded participants did not vary significantly from the population means. Participants were grouped as omnivores (OMN: n = 78) or vegetarians (VEG: n = 60). VEG were defined as participants who excluded all flesh foods. Of the OMN participants, 82% were at least monthly fish-eaters, and about one-third ate fish weekly.

Table 1 displays the participant characteristics by group. OMN participants were younger than VEG participants (41.00 ± 1.40 vs 45.07 ± 1.42 yrs, respectively; *p *= .046), their BMI was higher than VEG participants (27.82 ± 0.90 kg/m^2 ^vs 25.09 ± 0.72 kg/m^2^, *p *= .024), and their total physical activity level score was lower than VEG participants (22.12 ± 2.31 vs 32.08 ± 3.59, *p *= .016). Packed red cell volume, assessed on a subsample of participants (n = 62), did not differ between diet groups (42.14 ± 0.62 vs 42.83 ± 0.88 *p *= .519).

**Table 1:** Characteristics of participants by diet group

	**OMN**		**VEG**		**P**
	**Mean**	**± SE**	**Mean**	**± SE**	**value**
	**n = 78**		**n = 60**		*
		
Age	41.00	1.40	45.07	1.42	0.046
Gender M/F, n/n	33/45		28/32		0.609
Body Mass Index, kg/m2	27.82	0.90	25.09	0.72	0.024
Total PA level (1)	22.12	2.31	32.08	3.59	0.016
Attended college, %	35		48		0.104

* < 0.05 is significant.

1 Total weekly leisure activity scores - sum of frequencies of activity intensities (see Methods)

### Fatty acid intakes

Compared to OMN participants, VEG participants reported significantly lower EPA (0.005 ± 0.004 g vs 0.093 ± 0.027 g; *p *< .001), DHA (0.015 ± 0.007 g vs 0.162 ± 0.040 g; *p *< .001), and AA (0.011 ± 0.003 g vs 0.086 ± 0.011 g; *p *< .001) intakes. Table 2 displays the fatty acid intakes reported by group. VEG participants also reported significantly higher ALA (2.86 ± 0.42 g vs 1.48 ± 0.28 g; *p *< .001) and LA (14.83 ± 1.30 g vs 9.16 ± 0.85 g; p < .001) compared to OMN participants. There were no nutrient intake differences by gender (data not shown).

**Table 2:** Fatty acid intakes of participants by diet group

	**OMN**	**VEG**	**P**
	**Mean ± SE**	**Mean ± SE**	**value**
	**g**	**g**	*
		
α-Linolenic	1.48 ± 0.28	2.86 ± 0.42	< 0.001
Eicosapentaenoic	0.09 ± 0.03	0.01 ± 0.00	< 0.001
Docosahexaenoic	0.16 ± 0.04	0.02 ± 0.01	< 0.001
Total n-3	1.83 ± 0.31	3.04 ± 0.43	< 0.001
Linoleic	9.17 ± 0.85	14.83 ± 1.30	< 0.001
Arachidonic	0.09 ± 0.01	0.01 ± 0.00	< 0.001
Total n-6	9.49 ± 0.88	15.09 ± 1.35	< 0.001
Total polyunsaturated	11.34 ± 1.08	18.17 ± 1.59	< 0.001
Monounsaturated	14.99 ± 1.30	18.24 ± 1.36	0.017
Saturated	8.91 ± 0.66	7.94 ± 0.63	0.402
Total fatty acids	35.24 ± 2.81	44.34 ± 3.20	0.008
n-6/n-3, g/g	9.13 ± 0.64	8.18 ± 0.71	0.306

* < 0.05 is significant.

### Mood scores

Mean DASS and POMS scores by group are presented in Table 3. Mean total DASS scores of VEG participants were significantly lower than OMN participants (8.32 ± 0.88 vs 17.52 ± 1.88, *p *< .001), as were all three mean subscales scores, indicating better mood in VEG participants (Table 3). When adjusting for diet group, there was no significant difference in mean total DASS scores between males and females (10.69 ± 1.22 vs 15.75 ± 1.88, respectively; *p *= .116), and PA level explained about 6% of the variance in mean total DASS scores (*p *= .004).

**Table 3:** DASS and POMS scores by diet group

	**OMN**		**VEG**		**P**
	**Mean**	**± SE**	**Mean**	**± SE**	**value**
	**n = 78**		**n = 60**		*
		
DASS-total^1^	17.51	1.88	8.32	0.88	0.000
DASS-D	4.81	0.69	1.67	0.28	0.000
DASS-A	4.31	0.53	1.53	0.24	0.000
DASS-S	8.40	0.92	5.12	0.52	0.024
					
POMS-total^2^	15.33	3.10	0.10	1.99	0.007
Tension-anxiety	6.04	3.83	3.83	0.40	0.031
Depression-dejection	8.99	0.80	4.36	4.10	0.000
Anger-hostility	7.08	6.72	4.28	0.55	0.010
Fatigue	7.59	0.66	5.03	0.47	0.021
Confusion	4.65	0.43	3.24	0.38	0.085
Vigor	19.15	0.71	20.61	0.71	0.133

* < 0.05 is significant.

1 DASS normative scores: D-5.55, A-3.56, S-9.27, total 18.38.

2 POMS normative scores, M-F: T 7.1-8.2, D 7.5-8.5, A 7.1-8.0, V 19.8-18.9, F 7.3-8.7, C 5.6-5.8, total 14.8-20.3.

**Figure 1:**
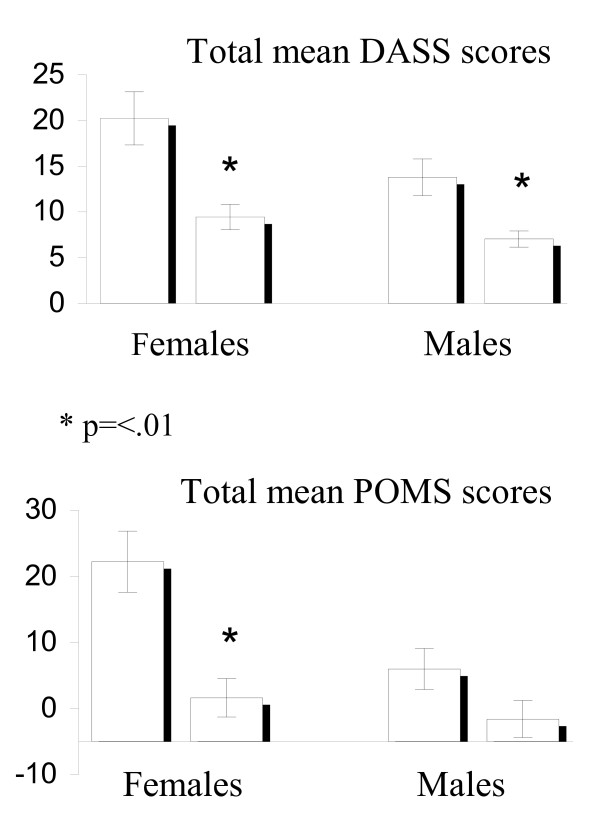
**Total mean mood scores by gender**.

Mean total POMS scores of VEG participants were also significantly lower than OMN participant scores (0.10 ± 1.99 vs 15.33 ± 3.10, *p *= .007), as were the domain scores except for POMS-C and POMS-V, indicating better mood in VEG participants (Table 3). It should be noted that gender impacted the total POMS scores, with females scoring significantly higher than males (13.62 ± 3.17 vs 2.51 ± 2.15, *p *< .001). When mean total POMS scores were analyzed by gender, controlling for covariates, there was no difference in scores between OMN and VEG among males (*p *= .934), but OMN females scored substantially higher than VEG females (22.18 ± 4.64 vs 1.59 ± 2.86, *p *= .005). PA level explained about 20% of the variance (*p *= .003). Figure 1 compares total mean DASS and POMS scores for VEG and OMN participants by gender.

Total mean DASS and POMS scores in the total sample were positively related to dietary intakes of EPA (*rho *= .259 and .278; *p *< 0.05), DHA (*rho *= .265 and .286; *p *< 0.05), and AA (*rho *= .284 and .331; *p *< 0.05), and inversely related to intakes of ALA (*rho *= -.227 and -.184; *p *< 0.05), and LA (*rho *= -.194 and -.249; *p *< 0.05), indicating that participants with low intakes of EPA, DHA, and AA and high intakes of ALA and LA had better mood. When correlations were examined in VEG and OMN participants separately, only AA intake and total mean POMS scores in OMN participants were positively correlated (*rho *= .256, *p *= .024). There was a strong, positive correlation between total mean DASS and POMS scores in the total sample (*rho *= .749, n = 138, *p *= .000). Total mean POMS scores were inversely related to age (*rho *= -.200, *p *= .020) and total mean DASS and POMS scores were inversely related to total PA levels (*rho *= -.265, p = .002; *rho *= -.231, *p *= .006, respectively) in the total sample.

## Discussion

Despite significantly lower reported EPA and DHA intakes, the vegetarians in our survey reported significantly less negative emotion than the omnivores as measured by both mood scales. Based on published POMS normative scores (total 14.8 to 20.3)[26], the VEG participants reported a more favorable mood state relative to OMN participants (0.10 ± 1.99 vs 15.33 ± 3.10 respectively, *p *= .007). Interestingly, recent investigations in healthy adult populations directly link POMS scores (range for mean population scores in these trials, 1.5 to 7.0) with markers of poor health outcomes including reduced flow-mediated dilation, metabolic syndrome, and job-related stress [29-31]. This interrelationship between health, mood, and diet patterns deserves further study. The DASS scores also indicated a more favorable mood state for VEG participants as compared to OMN participants (8.32 ± 0.88 vs 17.52 ± 1.88, *p *= .000), thus DASS and POMS data clearly complement each other, providing evidence that vegetarian diets are not likely associated with poor mood states or depression as compared to omnivorous diets, despite negligible sources of the long-chain omega-3 fats.

These results challenge what is known about the link between dietary fats and brain function and suggest an unrecognized benefit of vegetarian diets which are naturally low in the long-chain omega 3 fats. Several randomized, placebo-controlled trials have demonstrated beneficial effects of fish or fish oil on mood states as indicated by significant reductions in POMS scores in healthy volunteers [32,33]. In these subject populations, markers of oxidative stress were reduced in the fish-supplemented groups concomitant with improved mood states, findings that support earlier descriptive data directly linking oxidative stress and psychological distress [34]. Vegetarians are generally characterized by high circulating concentrations of antioxidants and reduced oxidative stress [35,36], attributed to high intakes of plant foods in this population. Markers of oxidative stress were not collected in the present trial, and mechanisms to explain associations between vegetarian diets and improved mood states have not been explored.

The vegetarians reported significantly higher LA and ALA intakes than the omnivores, a profile well established in the literature [37]. High intakes of these polyunsaturated fatty acids have been shown to inhibit the activity of desaturases required for endogenous formation of longer chain metabolites; thus, as blood levels of LA rise, levels of AA reportedly do not, and inflammation is decreased [38-40]. Moreover, absolute amounts of LA and ALA have been shown to be more important than relative proportions of these fatty acids when it comes to conversion efficiency [41,42]. Based on his latest review of published data on dietary intake of polyunsaturated fatty acids by vegetarians, Sanders [43] concluded that plasma proportions of EPA and DHA may be adequate in vegetarians as long as there is high ALA intake which lowers the LA/ALA ratio. A number of studies that compared dietary fat intake and blood lipids of omnivores and vegetarians found that AA content of serum lipids was either similar or significantly lower in vegetarians than in the omnivores despite higher LA concentrations and lower EPA and DHA concentrations [3,4,6,11,44,45]. Thus, perhaps low intakes of EPA and DHA are not linked to adverse mood state in vegetarian populations, because intakes of ALA are generally high, AA intakes are low, and conversion of LA to AA is regulated.

A major limitation of our study was not measuring blood fatty acid concentrations or inflammatory markers. However, the use of food frequency questionnaires is considered effective in capturing omega-3 fatty acid intake since sources are consumed relatively infrequently and can be reported fairly accurately [46]. Our data may have been influenced by response bias, since SDA vegetarians may be more defensive about their diet choice than SDA omnivores, however, participants were not aware that the focus of the study was on vegetarian diets. Also, vegetarians may make better dietary choices, and may generally be healthier and happier [17]. In exclusively surveying the SDA community, we were able to identify vegetarian participants and analyze a relatively homogenous population of vegetarians and omnivores, thus minimizing potentially confounding lifestyle differences. These results, however, may not be generalizable to non-SDA populations.

## Conclusions

While dietary intake of EPA and DHA has an important role in brain function, we found no evidence that the absence of direct intake of these fatty acids in vegetarians adversely affects mood state. Features of the vegetarian diet profile such as higher intake of total polyunsaturated fat and negligible arachidonic acid intake may help explain the favorable mood profile we observed with vegetarian diets. Future research exploring the effect of dietary fat modifications on omnivore mental health may have public health importance.

## Competing interests

The authors declare that they have no competing interests.

## Authors' contributions

BLB participated in the study design and acquisition of data, performed the statistical analysis and interpretation of results, and drafted the manuscript. CSJ participated in the study design and acquisition of data, assisted in the statistical analysis and interpretation of results, and edited the manuscript. DRD participated in the dietary data analysis. All authors read and approved the final manuscript.

## References

[B1] Key TJ Fraser GE Thorogood M Appleby PN Beral V Reeves G Burr ML Chang-Claude J Frentzel-Beyme R Kuzma JW Mann J McPherson K Mortality in vegetarians and nonvegetarians: detailed findings from a collaborative analysis of 5 prospective studies Am J Clin Nutr 1999 70 516S 524S 10479225 10.1093/ajcn/70.3.516s

[B2] Freeman MP Hibbeln JR Wisner KL Davis JM Mischoulon D Peet M Keck PE Marangell LB Richardson AJ Lake J Stoll AL Omega-3 fatty acids: evidence basis for treatment and future research in psychiatry J Clin Psychiatry 2006 67 1954 1967 10.4088/JCP.v67n1217 17194275

[B3] Kornsteiner M Singer I Elmadfa I Very low n-3 long-chain polyunsaturated fatty acid status in Austrian vegetarians and vegans Ann Nutr Metab 2008 52 37 47 10.1159/000118629 18305382

[B4] Rosell MS Lloyd-Wright Z Appleby PN Sanders TA Allen NE Key TJ Long-chain n-3 polyunsaturated fatty acids in plasma in British meat-eating, vegetarian, and vegan men Am J Clin Nutr 2005 82 327 334 16087975 10.1093/ajcn.82.2.327

[B5] Lee HY Woo J Chen ZY Leung SF Peng XH Serum fatty acid, lipid profile and dietary intake of Hong Kong Chinese omnivores and vegetarians Eur J Clin Nutr 2000 54 768 773 10.1038/sj.ejcn.1601089 11083485

[B6] Agren JJ Tormala ML Nenonen MT Hanninen OO Fatty acid composition of erythrocyte, platelet, and serum lipids in strict vegans Lipids 1995 30 365 369 10.1007/BF02536047 7609607

[B7] Haag M Essential fatty acids and the brain Can J Psychiatry 2003 48 195 203 12728744 10.1177/070674370304800308

[B8] Calder PC N-3 polyunsaturated fatty acids and inflammation: from molecular biology to the clinic Lipids 2003 38 343 352 10.1007/s11745-003-1068-y 12848278 PMC7101988

[B9] Farooqui AA Horrocks LA Farooqui T Modulation of inflammation in brain: a matter of fat J Neurochem 2007 101 577 599 10.1111/j.1471-4159.2006.04371.x 17257165

[B10] Stahl LA Begg DP Weisinger RS Sinclair AJ The role of omega-3 fatty acids in mood disorders Curr Opin Investig Drugs 2008 9 57 64 18183532

[B11] Phinney SD Odin RS Johnson SB Holman RT Reduced arachidonate in serum phospholipids and cholesteryl esters associated with vegetarian diets in humans Am J Clin Nutr 1990 51 385 392 2106775 10.1093/ajcn/51.3.385

[B12] Burdge GC Metabolism of alpha-linolenic acid in humans Prostaglandins Leukot Essent Fatty Acids 2006 75 161 168 10.1016/j.plefa.2006.05.013 16828546

[B13] Angela Liou Y Innis SM Dietary linoleic acid has no effect on arachidonic acid, but increases n-6 eicosadienoic acid, and lowers dihomo-gamma-linolenic and eicosapentaenoic acid in plasma of adult men Prostaglandins Leukot Essent Fatty Acids 2009 80 201 206 10.1016/j.plefa.2009.02.003 19356914

[B14] Cleland LG James MJ Neumann MA D'Angelo M Gibson RA Linoleate inhibits EPA incorporation from dietary fish-oil supplements in human subjects Am J Clin Nutr 1992 55 395 399 1310374 10.1093/ajcn/55.2.395

[B15] Gronn M Gorbitz C Christensen E Levorsen A Ose L Hagve TA Christophersen BO Dietary n-6 fatty acids inhibit the incorporation of dietary n-3 fatty acids in thrombocyte and serum phospholipids in humans: a controlled dietetic study Scand J Clin Lab Invest 1991 51 255 263 10.3109/00365519109091612 1909049

[B16] Montgomery S Herring P Yancey A Beeson L Butler T Knutsen S Sabate J Chan J Preston-Martin S Fraser G Comparing self-reported disease outcomes, diet, and lifestyles in a national cohort of black and white Seventh-day Adventists Prev Chronic Dis 2007 4 A62 17572966 PMC1955428

[B17] Fraser GE Diet as primordial prevention in Seventh-Day Adventists Prev Med 1999 29 S18 23 10.1006/pmed.1998.0415 10641813

[B18] Godin G Shephard RJ A simple method to assess exercise behavior in the community Can J Appl Sport Sci 1985 10 141 146 4053261

[B19] Knechtle B Influence of physical activity on mental well-being and psychiatric disorders Praxis (Bern 1994) 2004 93 1403 1411 10.1024/0369-8394.93.35.1403 15468581

[B20] Maes M Song C Lin A De Jongh R Van Gastel A Kenis G Bosmans E De Meester I Benoy I Neels H Demedts P Janca A Scharpe S Smith RS The effects of psychological stress on humans: increased production of pro-inflammatory cytokines and a Th1-like response in stress-induced anxiety Cytokine 1998 10 313 318 10.1006/cyto.1997.0290 9617578

[B21] Lovibond PF Lovibond SH The structure of negative emotional states: comparison of the Depression Anxiety Stress Scales (DASS) with the Beck Depression and Anxiety Inventories Behav Res Ther 1995 33 335 343 10.1016/0005-7967(94)00075-U 7726811

[B22] Brown TA Chorpita BF Korotitsch W Barlow DH Psychometric properties of the Depression Anxiety Stress Scales (DASS) in clinical samples Behav Res Ther 1997 35 79 89 10.1016/S0005-7967(96)00068-X 9009048

[B23] Crawford JR Henry JD The Depression Anxiety Stress Scales (DASS): normative data and latent structure in a large non-clinical sample Br J Clin Psychol 2003 42 111 131 10.1348/014466503321903544 12828802

[B24] Crawford JR Garthwaite PH Lawrie CJ Henry JD MacDonald MA Sutherland J Sinha P A convenient method of obtaining percentile norms and accompanying interval estimates for self-report mood scales (DASS, DASS-21, HADS, PANAS, and sAD) Br J Clin Psychol 2009 48 163 180 10.1348/014466508X377757 19054433

[B25] Cranford JA Shrout PE Iida M Rafaeli E Yip T Bolger N A procedure for evaluating sensitivity to within-person change: can mood measures in diary studies detect change reliably? Pers Soc Psychol Bull 2006 32 917 929 10.1177/0146167206287721 16738025 PMC2414486

[B26] Nyenhuis DL Yamamoto C Luchetta T Terrien A Parmentier A Adult and geriatric normative data and validation of the profile of mood states J Clin Psychol 1999 55 79 86 10.1002/(SICI)1097-4679(199901)55:1<79::AID-JCLP8>3.0.CO;2-7 10100834

[B27] Baines S Powers J Brown WJ How does the health and well-being of young Australian vegetarian and semi-vegetarian women compare with non-vegetarians? Public Health Nutr 2007 10 436 442 10.1017/S1368980007217938 17411462

[B28] Benton D Donohoe RT The effects of nutrients on mood Public Health Nutr 1999 2 403 409 10.1017/S1368980099000555 10610080

[B29] Cooper DC Milic MS Tafur JR Mills PJ Bardwell WA Ziegler MG Dimsdale JE Adverse impact of mood on flow-mediated dilation Psychosom Med 2010 72 122 127 10.1097/PSY.0b013e3181cdbfc0 20100885 PMC3163844

[B30] Takeuchi T Nakao M Nomura K Inoue M Tsurugano S Shinozaki Y Yano E Association of the metabolic syndrome with depression and anxiety in Japanese men: a 1-year cohort study Diabetes Metab Res Rev 2009 25 762 767 10.1002/dmrr.1041 19839027

[B31] Fukuda H Ichinose T Kusama T Yoshidome A Anndow K Akiyoshi N Shibamoto T The relationship between job stress and urinary cytokines in healthy nurses: a cross-sectional study Biol Res Nurs 2008 10 183 191 10.1177/1099800408323219 18829600

[B32] Fontani G Corradeschi F Felici A Alfatti F Bugarini R Fiaschi AI Cerretani D Montorfano G Rizzo AM Berra B Blood profiles, body fat and mood state in healthy subjects on different diets supplemented with Omega-3 polyunsaturated fatty acids Eur J Clin Invest 2005 35 499 507 10.1111/j.1365-2362.2005.01540.x 16101670

[B33] Nozawa Y Ishizaki T Kuroda M Noguchi T Effect of dried-bonito broth intake on peripheral blood flow, mood, and oxidative stress marker in humans Physiol Behav 2008 93 267 273 10.1016/j.physbeh.2007.08.021 17945318

[B34] Irie M Asami S Nagata S Miyata M Kasai H Psychological mediation of a type of oxidative DNA damage, 8-hydroxydeoxyguanosine, in peripheral blood leukocytes of non-smoking and non-drinking workers Psychother Psychosom 2002 71 90 96 10.1159/000049351 11844945

[B35] Szeto YT Kwok TC Benzie IF Effects of a long-term vegetarian diet on biomarkers of antioxidant status and cardiovascular disease risk Nutrition 2004 20 863 866 10.1016/j.nut.2004.06.006 15474873

[B36] Krajcovicova-Kudlackova M Valachovicova M Paukova V Dusinska M Effects of diet and age on oxidative damage products in healthy subjects Physiol Res 2008 57 647 651 17705666 10.33549/physiolres.931244

[B37] Key TJ Appleby PN Rosell MS Health effects of vegetarian and vegan diets Proc Nutr Soc 2006 65 35 41 10.1079/PNS2005481 16441942

[B38] Adam O Tesche A Wolfram G Impact of linoleic acid intake on arachidonic acid formation and eicosanoid biosynthesis in humans Prostaglandins Leukot Essent Fatty Acids 2008 79 177 181 10.1016/j.plefa.2008.09.007 18973995

[B39] Zhao G Etherton TD Martin KR West SG Gillies PJ Kris-Etherton PM Dietary alpha-linolenic acid reduces inflammatory and lipid cardiovascular risk factors in hypercholesterolemic men and women J Nutr 2004 134 2991 2997 15514264 10.1093/jn/134.11.2991

[B40] Adam O Beringer C Kless T Lemmen C Adam A Wiseman M Adam P Klimmek R Forth W Anti-inflammatory effects of a low arachidonic acid diet and fish oil in patients with rheumatoid arthritis Rheumatol Int 2003 23 27 36 12548439 10.1007/s00296-002-0234-7

[B41] Goyens PL Spilker ME Zock PL Katan MB Mensink RP Conversion of alpha-linolenic acid in humans is influenced by the absolute amounts of alpha-linolenic acid and linoleic acid in the diet and not by their ratio Am J Clin Nutr 2006 84 44 53 16825680 10.1093/ajcn/84.1.44

[B42] Goyens PL Spilker ME Zock PL Katan MB Mensink RP Compartmental modeling to quantify alpha-linolenic acid conversion after longer term intake of multiple tracer boluses J Lipid Res 2005 46 1474 1483 10.1194/jlr.M400514-JLR200 15834128

[B43] Sanders TA DHA status of vegetarians Prostaglandins Leukot Essent Fatty Acids 2009 81 137 41 10.1016/j.plefa.2009.05.013 19500961

[B44] Fisher M Levine PH Weiner B Ockene IS Johnson B Johnson MH Natale AM Vaudreuil CH Hoogasian J The effect of vegetarian diets on plasma lipid and platelet levels Arch Intern Med 1986 146 1193 1197 10.1001/archinte.146.6.1193 3718107

[B45] Li D Sinclair A Mann N Turner A Ball M Kelly F Abedin L Wilson A The association of diet and thrombotic risk factors in healthy male vegetarians and meat-eaters Eur J Clin Nutr 1999 53 612 619 10.1038/sj.ejcn.1600817 10477247

[B46] Sontrop J Campbell MK Omega-3 polyunsaturated fatty acids and depression: a review of the evidence and a methodological critique Prev Med 2006 42 4 13 10.1016/j.ypmed.2005.11.005 16337677

